# Exploring Memories of the Self: 2412 Self-image Norms for Adults Aged 17 to 88

**DOI:** 10.3389/fpsyg.2017.01445

**Published:** 2017-08-23

**Authors:** Clare J. Rathbone, Chris J. A. Moulin

**Affiliations:** ^1^Department of Psychology, Social Work and Public Health, Oxford Brookes University Oxford, United Kingdom; ^2^LPNC, Centre National de la Recherche Scientifique UMR 5015, Université Pierre Mendès France Grenoble, France

**Keywords:** self-images, identity, self, autobiographical memory, personality

## Introduction

Self-images are “I am” statements (such as “I am an optimist”). They are frequently used in psychological research as a measure of identity, as primes in experimental tasks, and as cues to elicit autobiographical memories. Typically, participants are simply asked to answer the question “Who am I?,” such as in the 20 Statements Test (Kuhn and McPartland, 1954) which asks participants to generate up to 20 sentences starting with the words “I am.” A more recent modification of this approach (the IAM Task; Rathbone et al., 2008) asks participants for between 4 and 10 “I am” statements and then uses these as autobiographical memory cues. Self-images can include traits (e.g., I am happy), roles (e.g., I am a nurse), hobbies (e.g., I am a tennis player), hopes (e.g., I am keen to work with animals), and fears (e.g., I am scared of being lonely). They comprise semantic facts about the self which help to scaffold narrative continuity and reflect the multi-faceted nature of the self (Kuhn and McPartland, 1954; Markus, 1977), allowing researchers to tap into the multiple identities people possess. One important benefit of self-image generation tasks is their open-ended nature. Tasks involving the completion of “I am” statements allow people to describe their identity using their own words. As a result, they are a powerful tool for examining differences in how identity is constructed across different groups.

Studies from a range of psychological disciplines have analyzed “I am” statements. From a social perspective, researchers have analyzed differences in self-images across age (McCrae and Costa, 1988), gender (Grace and Cramer, 2002), profession (Kuhn, 1960), and culture (Rhee et al., 1995; Wang, 2001). Within cognitive psychology, self-images have been used in experiments, both to prime a particular cultural identity (Wang, 2008) and to measure self-concept following an experimental memory manipulation (Charlesworth et al., 2015; Çili and Stopa, 2015). They have also been used as autobiographical memory cues (Rathbone et al., 2008, 2015). With clinical relevance, self-images have been explored in relation to psychopathological disorders including PTSD (Abdollahi et al., 2012) and schizophrenia (Bennouna-Greene et al., 2012). Self-images have also been studied in neuropsychological patients with amnesia (Rathbone et al., 2009; Grilli and Verfaellie, 2015) and Alzheimer's disease (Addis and Tippett, 2004), in an effort to understand the impact of memory loss on the self. As is evident from this brief review, self-images are relevant to a wide range of disciplines and have been utilized in a variety of paradigms.

The purpose of this paper is to present a database of self-image norms. At present, to the authors' knowledge, there is no publicly available resource for collating or depositing self-image data. That also means, that aside of attempts to classify the statements generated [e.g., into consensual and non-consensual (Kuhn and McPartland, 1954); concrete or abstract (Rathbone et al., 2008); psychological, social, and physical (Charlesworth et al., 2015)], there has been little consideration of the content of people's identity statements. Whilst coding schemes have been developed in the past to classify responses (Cousins, 1989; Rhee et al., 1995), these have been limited to a relatively small number of categories (e.g., a maximum of 45 categories and subcategories; Rhee et al., 1995) and thus do not provide information on the frequency of specific self-images (e.g., being logical). Furthermore, previous studies have not reported the gender ratio or mean age of categories of self-images. Having a database of normative responses, coded by age and gender, will mean we will be better able to consider the content in people's identity statements.

The norms presented here represent the coding of 2,412 self-images. However, the main aim here is for this number to increase as other researchers add their data to the database. These norms can be used in a variety of ways. For example, if self-images have been collected from a case study (as is common in memory impairment) or a specific group of participants and the aim is to examine the way these participants have defined themselves, then the norms in the database can provide an age and gender indexed comparison. This will provide some information on whether a particular group of participants have described their identity in a way that is typical or atypical for their age and gender. This would be particularly relevant to studying possible changes to the self in clinical groups or patient case studies. At present, open-ended measures of self are limited to probing the number of self-images a person can generate, or the abstract/specific nature of these self-images (Addis and Tippett, 2004).

In addition to its utility in clinical groups, these norms will also be of use for researchers who want to use them as experimental primes or cues. Having identified which self-images are most commonly generated in young adults, for example, these could be used as standardized memory cues or primes in cognitive tasks, in the same way that it is common to control for word frequency or concreteness when using words as stimuli. As such, this database will provide a useful resource for cognitive and social psychologists, as well as sociologists and anthropologists. Finally, by tagging the year and country of data collection, as the database grows it will be possible to track changes in identity descriptions over time and across different nationalities.

## Methods and materials

### Participants

The data come from five studies, all of which required participants to generate self-images. Studies 1 and 2 are reported in Rathbone et al. (2008). Study 1 had an age range of 47–66 (*N* = 16 [11 Female], Mean age 54.56, SD = 4.95) and Study 2 had an age range of 39–88 (*N* = 125 [81 Female], Mean age 52.94, SD = 10.19). Note that for Study 2 the sample size in the database is higher than in Study 2 of Rathbone et al. (2008) as we only reported data from participants who completed the online questionnaire. In the database we include self-images for all participants who generated at least one self-image, leading to the discrepancy between sample sizes. Study 3 (reported in Rathbone and Moulin, 2014) had an age range of 17–50 (*N* = 40 [33 Female], Mean age 19.63, SD = 5.01). Studies 4 and 5 (Rathbone and Moulin, 2017) had respective age ranges of 19–61 (*N* = 40 [30 Female], Mean age 24.2, SD = 8.63) and 19–34 (*N* = 17 [12 Female], Mean age 20.56, SD = 3.65). These data were collected between 2004 and 2008, using an online questionnaire (Study 2) and paper questionnaires/tasks completed in participants' own homes (Study 1) or in the lab (Studies 3, 4, and 5). The data from studies employing paper questionnaires was collected in the UK whilst the online questionnaire data had potential for worldwide geographical coverage (details of participant nationality were not collected but the online questionnaire was advertised in both the UK and USA).

### Task

All participants were asked to generate a set of between 4 and 20 “I am” statements to describe long-term and enduring aspects of their identity (for the full protocol see Rathbone et al., 2008). In Study 5 (*N* = 17), the instructions differed slightly as participants were asked to generate five self-images associated with being at home, and five associated with being at university. In all other cases, participants were free to generate whichever statements they felt best defined their identity.

### Coding

All 2,412 self-images were coded by CJR and CJAM. This generated a set of 271 categories (including “Unclassified” which referred to any category indexing only one self-image). All 270 categories which indexed two or more self-images were then listed as norms (see Supplementary Material), accompanied by the number of self-images in the category, the full-text of each self-image generated, and the mean age and gender ratio of the participants that generated self-images in that category. For example, “Planner” had a count of three (two cases of “a planner” and one case of “able to plan ahead”), a mean age 28.67, and was only generated by female participants. Due to the way in which data was processed it is possible in a few cases that there is a discrepancy between the age given in the self-image and the participant's noted age. In one case this is due to a participant reporting an age which is actually different from their chronological age. For other cases it is due to how the data was processed by calculating the participant's age from a given year of birth. If a participant generated a self-image that covered more than one category then only the first category was coded (e.g., “Caring and sensitive” was coded as “Caring,” “a daughter and sister” was coded as “Daughter,” and “Independent, intelligent, adaptable, love travel” was coded as “Independent”). Our decision to code the first self-image in cases where two or more were generated was empirically grounded and based on previous work which shows that the self-images people generate first are generally rated as more personally significant (Rathbone and Moulin, 2014).

### Database websites

The fixed data set is publicly available in the UK Data Service ReShare Repository under Collection A of “Memory and the self in ageing” (Rathbone, 2017) at: https://dx.doi.org/10.5255/UKDA-SN-852127. The data set includes a data description document which explains the variables in the excel spreadsheet. There is also a publicly accessible database website hosted by Oxford Brookes University (http://psych.brookes.ac.uk/selfimage) which provides an introduction to the database, a description of its potential uses, a search function, and the option to request a downloadable excel version of the database. One of the primary aims of the website is to form a long-term, growing resource for researchers who wish to deposit their self-image data. Thus, whilst the original data set, as reported in this paper, is fixed, there is also the option for this database to grow via the self-image website. Instructions are provided online for those who wish to upload data to the database. All uploaded data will be checked before being made publically available in the database.

### Ethics statement

All studies through which data was collected were approved by the University of Leeds School of Psychology Ethics Committee and were conducted according to the principles expressed in the Declaration of Helsinki. Any identifying information (such as participants' own names or the names of their friends, and of places) was removed during the preparation of the database. Written informed consent was obtained from participants in studies 3, 4, and 5. One participant in Study 3 was aged 17, but was deemed able to give informed consent without the need for additional consent from a next of kin, caretaker, or guardian. This was because the participant was enrolled as an undergraduate on a university degree course and because the British Psychological Society Code of Human Research Ethics classes participants aged 17 and over as a non-vulnerable group in terms of age. As studies 1 and 2 were solely questionnaire-based, participants were informed that submitting their data for analysis was taken as their consent to participate. Any new data submitted to the database will only be uploaded once ethical information has been provided.

## Analysis and interpretation

Full details of self-image norms are provided in the Supplementary Material. By examining the frequency counts we can see which categories are most often used when people describe their identities. The most commonly generated category of self-image was Friend (count = 77), followed by Student (count = 70), Sports player (count = 70), and Occupation (count = 68). Thus, specific social roles are the most commonly generated single categories across the database. The highest frequency traits were Happy (count = 43), Optimistic (count = 41), and Friendly (count = 38). In line with previous research showing that people tend to define themselves in broadly positive terms (e.g., Baumeister, 1998), the highest frequency categories of self-image traits are generally positive in valence. However, several negative self-images were also generated multiple times (e.g., Lazy = 19; Worry = 15; Pessimistic = 10). In terms of mean age of participants, the youngest self-image category generated was Maths (e.g., ok at maths; Mean age = 18) and the oldest was Serious (e.g., serious minded; Mean age = 67.5).

By probing the database as a whole, it is possible to identify the most frequently cited self-images for people in given age ranges or for specific genders. For example, within females aged 60–70 the most commonly cited category of self-image was Mother (*n* = 9). As well as providing a useful resource for researchers interested in the lifespan development of identity (by showing which identities are most relevant at different ages), these age-specific norms may have particular utility for matching to specific clinical samples. Furthermore, this database may provide a useful resource for researchers who study cultural life scripts: representations of the timing of major transitional life events within a specific culture (Rubin and Berntsen, 2003; Berntsen and Rubin, 2004). Studies have shown that these normative life events are typically positive and reflect social landmarks such as graduation, marriage, and parenthood (Berntsen and Rubin, 2004). This database allows investigation of the age of participants who identify with particular life script categories, such as being a mother (mean age 51.79). Thus, whilst parenthood is a life script event that typically occurs in young adulthood, it nevertheless remains a salient self-image for adults across the lifespan.

One issue with the database, as it stands, is that some self-image categories feature relatively low counts. For example, Articulate is only generated twice, so the mean age for this category is not as meaningful as that for Caring (with a count of 21). Furthermore, at present, the participants in the database are mostly female younger adults. This must be borne in mind when making comparisons by age and gender. For example, the fact that Sister was generated 65 times and Brother only 12 does not necessarily indicate that males are less likely than females to frame their identity around their siblings—rather, this is probably a reflection of the gender ratio in the database. What is more informative is the within-category gender ratio data. For example, Travel had a relatively even split of 12 males and 13 females, whereas Worry was generated by 14 females and only 1 male.

These norms can be used as the basis for further studies on the salience of specific self-images. For example, one could investigate what proportion of people who are brothers actually generate brother as a self-image. This could be achieved by asking participants to complete an open-ended 20 statements task first, followed by responding yes/no to a standardized set of self-images based on the norms reported here. In this way it will be possible to explore which self-images are most salient for different groups, whilst also being able to account for which self-images are objectively present.

As with any self-reported data on personal identity, it is important to acknowledge the impact of environmental cues and social expectations on generation of self-images (see Markus and Kunda, 1986). Whilst this database cannot address the issue of how true (as opposed to confabulatory) or stable these self-images are, they nevertheless reflect an accurate snapshot of our participants' views of themselves at a given moment in time.

In conclusion, this database and table of norms represents the self-images that are most salient for a group of adults at the start of the twenty-first century. It provides an indication of which self-images are most relevant to participants of different ages and genders, and will provide a lasting resource for other researchers who wish to use these data or contribute their own.

## Author contributions

CR and CM jointly designed the studies that comprise this data collection. Data analysis and preparation of this manuscript were carried out by both CR and CM.

### Conflict of interest statement

The authors declare that the research was conducted in the absence of any commercial or financial relationships that could be construed as a potential conflict of interest.

## References

[B1] Abdollahi M. Moradi A. Hasani J. Jobson L. (2012). Investigating the relationships between autobiographical remembering, the self and posttraumatic stress disorder in individuals with HIV. Memory 20, 872–881. 10.1080/09658211.2012.703211 22871136

[B2] Addis D. R. Tippett L. J. (2004). Memory of myself: autobiographical memory and identity in Alzheimer's disease. Memory 12, 56–74. 10.1080/09658210244000423 15098621

[B3] Baumeister R. F. (1998). The self, in Handbook of Social Psychology, eds Gilbert D. T. Fiske S. T. Lindzey G. (New York, NY: McGraw-Hill), 680–740.

[B4] Bennouna-Greene M. Berna F. Conway M. A. Rathbone C. J. Vidailhet P. Danion J. M. (2012). Self-images and related autobiographical memories in schizophrenia. Conscious. Cogn. 21, 247–257. 10.1016/j.concog.2011.10.006 22040535

[B5] Berntsen D. Rubin D. C. (2004). Cultural life scripts structure recall from autobiographical memory. Mem. Cogn. 32, 427–442. 10.3758/BF03195836 15285126

[B6] Charlesworth L. A. Allen R. J. Havelka J. Moulin C. J. A. (2015). Who am I? Autobiographical retrieval improves access to self-concepts. Memory 14, 1–9. 10.1080/09658211.2015.1063667 26273724

[B7] Çili S. Stopa L. (2015). The retrieval of self-defining memories is associated with the activation of specific working selves. Memory 23, 233–253. 10.1080/09658211.2014.882955 24528183

[B8] Cousins S. D. (1989). Culture and selfhood in Japan and the U.S. J. Pers. Soc. Psychol. 56, 124–131. 10.1037/0022-3514.56.1.124

[B9] Grace S. Cramer K. L. (2002). Sense of self in the new millennium: male and female student responses to the TST. Soc. Behav. Pers. 30, 271–280. 10.2224/sbp.2002.30.3.271

[B10] Grilli M. D. Verfaellie M. (2015). Supporting the self-concept with memory: insight from amnesia. Soc. Cogn. Affect. Neurosci. 10, 1684–1692. 10.1093/scan/nsv056 25964501 PMC4666106

[B11] Kuhn M. H. (1960). Self-attitudes by age, sex, and professional training. Sociol. Q. 1, 39–54. 10.1111/j.1533-8525.1960.tb01459.x

[B12] Kuhn M. H. McPartland T. S. (1954). An empirical investigation of self-attitudes. Am. Sociol. Rev. 19, 68–76. 10.2307/2088175

[B13] Markus H. (1977). Self-schemata and processing information about self. J. Pers. Soc. Psychol. 35, 63–78. 10.1037/0022-3514.35.2.63

[B14] Markus H. Kunda Z. (1986). Stability and malleability of the self-concept. J. Pers. Soc. Psychol. 51, 858–866. 10.1037/0022-3514.51.4.858 3783430

[B15] McCrae R. R. Costa P. T. (1988). Age, personality, and the spontaneous self-concept. J. Gerontol. 43, S177–S185. 10.1093/geronj/43.6.S177 3183315

[B16] Rathbone C. J. (2017). Memory and the Self in Ageing. Colchester: UK Data Archive.

[B17] Rathbone C. J. Holmes E. A. Murphy S. E. Ellis J. A. (2015). Autobiographical memory and well-being in aging: the central role of semantic self-images. Conscious. Cogn. 33, 422–431. 10.1016/j.concog.2015.02.017 25781408

[B18] Rathbone C. J. Moulin C. J. A. (2014). Measuring autobiographical fluency in the self-memory system. Q. J. Exp. Psychol. 67, 1661–1667. 10.1080/17470218.2014.913069 24712916

[B19] Rathbone C. J. Moulin C. J. A. (2017). Switch costs in the self memory system. Q. J. Exp. Psychol. 70, 1063–1073. 10.1080/17470218.2015.1127398 26652022

[B20] Rathbone C. J. Moulin C. J. A. Conway M. A. (2008). Self-centred memories: the reminiscence bump and the self. Mem. Cogn. 36, 1403–1414. 10.3758/MC.36.8.1403 19015500

[B21] Rathbone C. J. Moulin C. J. A. Conway M. A. (2009). Autobiographical memory and amnesia: using conceptual knowledge to ground the self. Neurocase 15, 405–418. 10.1080/13554790902849164 19382038

[B22] Rhee E. Uleman J. S. Roman R. J. Lee H. K. (1995). Spontaneous self-descriptions and ethnic identities in individualistic and collectivistic cultures. J. Pers. Soc. Psychol. 69, 142–152. 10.1037/0022-3514.69.1.142 7643297

[B23] Rubin D. C. Berntsen D. (2003). Life scripts help to maintain autobiographical memories of highly positive, but not highly negative, events. Mem. Cogn. 31, 1–14. 10.3758/BF03196077 12699138

[B24] Wang Q. (2001). Culture effects on adults' earliest childhood recollection and self-description: implications for the relation between memory and the self. J. Pers. Soc. Psychol. 81, 220–233. 10.1037/0022-3514.81.2.220 11519928

[B25] Wang Q. (2008). Being American, being Asian: the bicultural self and autobiographical memory in Asian Americans. Cognition 107, 743–751. 10.1016/j.cognition.2007.08.005 17920579

