# Supplementary material for: Exploring Memories of the Self: 2412 Self-image Norms for Adults Aged 17 to 88
Source: Front Psychol. 2017 Aug 23;8:1445. doi: 10.3389/fpsyg.2017.01445 (PMC5572507; doi:10.3389/fpsyg.2017.01445)
Supplement: Supplementary file 1 [file Table1.pdf]

Supplementary Material

Exploring memories of the self: 2412 Self-image norms for adults aged 17 to 88

Clare J. Rathbone, Chris J.A. Moulin

*** Correspondence:** crathbone@brookes.ac.uk

Supplementary Data: Self-image norms with frequency, mean age (in years) and gender split

A LAUGH (2)
Mean age 21.5
Gender split 1 M; 1 F
A laugh (1), Up for a laugh (1)

ACADEMIC (5)
Mean age 32
Gender split 2 M; 3 F
Academic (3), Academically minded (1), Rather academic (1)

ACTIVE (7)
Mean age 36.71
Gender split 2 M; 5 F
Active (4), Active, love music, dancing, walking, I am a positive thinker (1), An active person (1), Fully active (1)

ADVENTUROUS (7)
Mean age 26.14
Gender split 2 M; 5 F
Adventurous (6), Someone who likes random adventures (1)

AFFECTIONATE (2)
Mean age 20
Gender split 2 F
Affectionate (2)

AFRAID (8)
Mean age 32.75
Gender split 1 M; 7 F
Afraid of confrontation (1), Afraid of not being liked (1), Afraid of pigs (1), Easily frightened (1), Scared easily (1), Scared of dogs (1), Scared of failing (1), Scared of the dark (1)

AGE (20)
Mean age 30.8
Gender split 6 M; 14 F
19 years old (2), 23 years old (1), Beginning to feel my age (1), Eighteen (3), Nineteen (3), Old (1), Over sixty (1), Twenty (3), Twenty-one (1), Twenty-five (1), Young (2), Young at heart (1)

ALCOHOL (6)
Mean age 18.83
Gender split 2 M; 4 F

A customer at the local pub (1), A drinker (2), A person who drinks at home (1), A person who enjoys relaxing with a glass of wine (1), A regular at the X Pub and X's (1)

AMBITIOUS (11)
Mean age 23.55
Gender split 2 M; 9 F

Ambitious (10), Ambitious, yet work to keep my life a good balance (1)

AMBIVERT (2)
Mean age 58
Gender split 2 F

Ambivert (1), An ambivert (1)

ANIMAL LOVER (6)
Mean age 33.33
Gender split 6 F

A dog lover (1), An animal lover (5)

ANIME (4)
Mean age 20
Gender split 1 M; 3 F

A member of anime society (1), An anime fanatic (1), An otaku (1), Anime obsessive (1)

ANXIOUS (5)
Mean age 35.2
Gender split 5 F

Usually very anxious about small things (1), Anxious (1), Anxious to "do things properly"(1), Quite an anxious person (1), Quite anxious (1)

APPEARANCE (4)
Mean age 48.5
Gender split 2 M; 2 F

Plain faced (not attractive- not ugly) (1), Pretty (1), Tanned (1), Wrinkly (1)

APPROACHABLE (5)
Mean age 19.6
Gender split 1 M; 4 F

Approachable (5)

ARGUMENTATIVE (3)
Mean age 21.67
Gender split 1 M; 2 F

Argumentative (3)

ART (10)
Mean age 36.8
Gender split 4 M; 6 F

A painter (1), An art lover (2), An artist (3), Artistic (1), Good at art (1), Good at drawing (1), Interested in art (1)

ARTICULATE (2)
Mean age 55.5
Gender split 2 F
Articulate (2)

ATHLECTIC (2)
Mean age 21
Gender split 1 M; 1 F
Athletic (2)

AUNT (5)
Mean age 24.6
Gender split 5 F
A aunt (1), A step-aunt (1), A wonderful auntie (1), An auntie (1), An aunty (1)

BALANCED (2)
Mean age 33.5
Gender split 1 M; 1 F
Balanced (2)

BORED (3)
Mean age 30
Gender split 1 M; 2 F
Bored with my lifestyle (1), Easily bored (2)

BOYFRIEND (2)
Mean age 22.5
Gender split 2 M
A boyfriend (2)

BROTHER (12)
Mean age 20.08
Gender split 12 M
A brother (10), An older brother (2)

BUBBLY (2)
Mean age 19
Gender split 2 F
Bubbly (2)

BUSY (2)
Mean age 39
Gender split 1 M; 1 F
Always busy (1), Someone who likes to feel busy (1)

CALM (4)
Mean age 19
Gender split 2 M; 2 F
Calm (4)

CAREER PLANS (4)
Mean age 36.25
Gender split 1 M; 3 F

Interested in working with people (1), Keen to be involved in developmental psychology of children (1), Keen to have a career I can take into retirement on a professional yet voluntary basis (1), Looking for a job (1)

CAREFUL (2)
Mean age 24
Gender split 2 F

Careful (2)

CARER (4)
Mean age 49.25
Gender split 4 F

A caregiver to my husband (1), A caregiver (1), A carer (2)

CARING (21)
Mean age 38.52
Gender split 4 M; 17 F

A caring person (1), Caring (16), Caring and sensitive (1), Caring to others (1), Someone who cares (1), Very caring for others' well-being (1)

CAUTIOUS (2)
Mean age 46
Gender split 1 M; 1 F

Cautious (2)

CHALLENGE (2)
Mean age 63.5
Gender split 2 F

Always ready to take up a challenge (1), Someone who likes a challenge (1)

CHATTING (3)
Mean age 19.33
Gender split 3 F

Chatty (2); Into chatting on MSN (1)

CHEERFUL (3)
Mean age 30.33
Gender split 1 M; 2 F

Cheerful (3)

CHILDLESS (2)
Mean age 46.5
Gender split 2 F

Childless (2)

CHRISTIAN (10)
Mean age 48.9

Gender split 4 M; 6 F

A Christian (5), A Christian – not organised religion though (1), A practicing Christian (1), Christian (3)

CLEAN (3)
Mean age 23
Gender split 1 M; 2 F

A clean person (1), Clean (2)

A CLUBBER (3)
Mean age 19
Gender split 3 F

A clubber (3)

COLLECTOR (2)
Mean age 61.5
Gender split 2 M

A collector (1), A collector and teller of stories and anecdotes (1)

COMMITTED (3)
Mean age 23
Gender split 1 M; 2 F

Committed (3)

COMPASSIONATE (3)
Mean age 53.67
Gender split 2 M; 1 F

Compassionate (3)

COMPETETIVE (2)
Mean age 21
Gender split 1 M; 1 F

Competitive (1), Quite competitive (1)

COMPLEX (2)
Mean age 40.5
Gender split 1 M; 1 F

A complex person (1), Complex (1)

CONCERNED (4)
Mean age 52
Gender split 1 M; 3 F

A concerned person (1), Concerned (1), Concerned about society (1), Concerned citizen (1)

CONFIDENT (20)
Mean age 28.55
Gender split 6 M; 14 F

A self-confident (1), Appear confident (1), Confident (16), Fairly confident (1), Self-confident (1)

CONSCIENTIOUS (5)
Mean age 21
Gender split 2 M; 3 F

Conscientious (5)

CONSIDERATE (4)
Mean age 29.75
Gender split 4 F
Considerate (4)

CONTENT (3)
Mean age 48.33
Gender split 1 M; 2 F
Content (1), Content in life (1), Contented (1)

CONTROL (2)
Mean age 40.5
Gender split 1 M; 1 F
Controlling (1), The type of person who likes to be in control (1)

COOKING (7)
Mean age 20.14
Gender split 2 M; 5 F
A bad cook (1), A cook (enjoy cooking) (1), A good cook (4), Average at cooking (1)

COUSIN (5)
Mean age 18.8
Gender split 5 F
A cousin (4), Cousin (1)

CRAZY (2)
Mean age 20
Gender split 2 F
Crazy (2)

CREATIVE (11)
Mean age 44.09
Gender split 1 M; 10 F
A creative thinker (1), Creative (10)

CURIOUS (13)
Mean age 38.92
Gender split 2 M; 11 F
A curious person (1), Curious (11), Curious about the world (1)

CYNICAL (3)
Mean age 29.67
Gender split 1 M; 2 F
Cynical (3)

DANCE (13)
Mean age 20.54
Gender split 1 M; 12 F

A bad dancer (1), A ballet dancer (1), A belly dancer (1), A dancer (6), A good dancer (1), A member of dance school (1), A member of dance street society (1), an Irish dancer (1)

DAUGHTER (66)
Mean age 24.08
Gender split 1 M; 65 F

A daughter (63), A daughter and sister (1), A step-daughter (1), Daughter (1)

DEDICATED (3)
Mean age 25.67
Gender split 2 M; 1 F

Dedicated (2), Dedicated to my beliefs (1)

DEPRESSION (3)
Mean age 31.33
Gender split 3 F

A depressive (1), Depressed sometimes (1), Suffering from a depressive disorder (1)

DETERMINED (11)
Mean age 30.82
Gender split 2 M; 9 F

Determined to make the most of my life (1), Determined (8), Determined to do well (1), Very determined (1)

DISCIPLINED (3)
Mean age 41
Gender split 1 M; 2 F

A disciplinarian (1), Disciplined (1), A disciplined person (1)

DISORGANISED (4)
Mean age 19.75
Gender split 4 F

Disorganised (2), Unorganised (2)

DISTRACTED (8)
Mean age 18.88
Gender split 3 M; 5 F

Distracted easily (1), Easily distracted (5), Fairly easily distracted (2)

DIVORCE (2)
Mean age 56.5
Gender split 1 M; 1 F

A reluctant divorcee (1), Twice divorced (1)

DOWN TO EARTH (2)
Mean age 33
Gender split 2 F

Down to earth (2)

DRAMATIC (2)
Mean age 18.5
Gender split 2 F

Dramatic (1), Over-dramatic (1)

DREAMER (2)
Mean age 39
Gender split 2 F
A dreamer (2)

DRIVING (12)
Mean age 22.92
Gender split 3 M; 9 F
A driver (9), A good driver (2), a motorist (1)

EASY GOING (15)
Mean age 19.8
Gender split 2 M; 13 F
Easy going (10), Easy to entertain (1), Easy to get along with (1), Easy to get on with (2), Easy to talk to (1)

EASY TO PLEASE (2)
Mean age 34.5
Gender split 2 F
Easily pleased (1), Easy to please (1)

EMOTIONAL (10)
Mean age 32.6
Gender split 4 M; 6 F
An emotional person (1), Bad in emotional situations (1), Emotional (6), Overly emotional (1), Very emotional (1)

EMPATHETIC (5)
Mean age 33.8
Gender split 5 F
Empathetic (4), Empathetic and a good listener (1)

ENERGETIC (8)
Mean age 32
Gender split 2 M; 6 F
Always full of energy (1), Energetic (7)

ENJOYMENT (3)
Mean age 35.67
Gender split 1 M; 2 F
Enjoying life (1), Enjoying my stay in X (1), Enjoying uni (1)

ENTHUSIASTIC (9)
Mean age 23.67
Gender split 9 F
Enthusiastic (9)

ENVIRONMENTALIST (2)
Mean age 56

Gender split 2 M

An environmentalist (1), Environmentalist (1)

ETHNICITY (6)
Mean age 30.67
Gender split 2 M; 4 F

African American (1), Arabic (1), Mixed race (1), White (2), White English (1)

EXCITED (7)
Mean age 24.14
Gender split 1 M, 6 F

Excitable (5), Excited (2)

EXTRAVERT (8)
Mean age 45
Gender split 4 M; 3 F; 1 unknown

An extravert (3), Extravert (2), Extraverted (2), Slightly extravert (1)

FAIR (2)
Mean age 34
Gender split 1 M; 1 F

Fair (1), Just and fair, a supporter of human rights for all (1)

FAMILY (22)
Mean age 36.36
Gender split 7 M; 15 F

A family man (3), A family person (3), A provider for my family (1), A regular visitor to my Mum's (1), A youngest child (2), Close to my family (2), Family oriented (1), Living with my family (1), Looking forward to seeing family (1), Part of a big family (1), Part of a family (1), Part of a loving family (1), The child of divorced parents (1), The head of the household (1), The hub of my family (1), The third child in family of ten (1)

FASHION (5)
Mean age 22
Gender split 5 F

A clothes-lover (1), Fashion-conscious (2), Interested in fashion (1), Oblivious to fashion (1)

FATHER (16)
Mean age 54.19
Gender split 16 M

Father (13), A father and grandfather (1), Father (2)

FEMINIST (3)
Mean age 59
Gender split 3 F

A feminist (3)

FITNESS (6)
Mean age 37.5
Gender split 2 M; 4 F

A physically fit person (1), Fit (2), Fit and an amateur gardener (1), Reasonably fit (1), Unfit (1)

FLATMATE (2)
Mean age 19
Gender split 1 M; 1 F
A flatmate (1), A member of a flat (1)

FOCUSED (2)
Mean age 22.5
Gender split 1 M; 1 F
Focused (2)

FOOD (6)
Mean age 26.5
Gender split 6 F
A chocoholic (1), A food lover (1), A person who likes eating at the chinese with X (1), Interested in healthy eating (1), Liking chocolate (1), Prone to comfort eating (1)

FORGETFUL (2)
Mean age 37
Gender split 2 F
Forgetful (2)

FORGIVING (3)
Mean age 33.67
Gender split 3 F
Forgiving (2), Quick to forgive (1)

FORTUNATE (2)
Mean age 19.5
Gender split 2 F
Fortunate (2)

FRIEND (77)
Mean age 23.55
Gender split 18 M; 59 F
A best friend (3), A close friend of X (1), A friend (26), A good friend (10), a loyal friend (1), A very good friend to have (1), Best friends with X (2), Close friends with X (8), Close friends with a group of girls (1), Close friends with old co-workers (1), Close to my friends (1), Enjoy the company of friends (1), Friend (1), Friends with X (9), Friends with (names) (1), Friends with a small group of girls (1), Good friends with X (2), Good friends with X and X (1), In a great circle of friends (1), Living with my mates (1), Looking for a few reliable 'real' friends (1), One who enjoys getting together with friends (1), Thankful for my friends (1), Very close to all my friends (1)

FRIENDLY (38)
Mean age 23.29
Gender split 9 M; 29 F
A friendly person (1), Friendly (36), Friendly person (1)

FUN (10)
Mean age 30.4
Gender split 1 M; 9 F
Fun (3), Fun loving (7)

FUNNY (11)
Mean age 25.27
Gender split 2 M; 9 F

Funny (10), Quite funny (1)

GAMES (2)
Mean age 22.5
Gender split 2 F

A gamer (1), Fond of games (1)

A GARDENER (3)
Mean age 54.33
Gender split 1 M; 2 F

A gardener (3)

GENDER (29)
Mean age 40.34
Gender split 9 M; 20 F

A boy (1), A female (3), A girl (6), A male (1), A man (1), A woman (5), A woman trapped in a man's body (1), A working woman (1), Female (6), Male (2), Transgender (1), Woman (1)

GENEROUS (6)
Mean age 37.33
Gender split 1 M; 5 F

Generous (6)

GENTLE (2)
Mean age 36
Gender split 2 M

A gentle man (1), Gentle (1)

GIRLFRIEND (24)
Mean age 21.13
Gender split 24 F

X's girlfriend (1), A girlfriend (18), A good girlfriend (1), An ex-girlfriend (2), Girlfriend (1), With my boyfriend (1)

GODMOTHER (2)
Mean age 21.5
Gender split 2 F

A godmother (2)

GOING OUT (7)
Mean age 21
Gender split 7 F

A person who goes out a lot (1), A person who goes out most nights (1), A person who goes out with friends from home (1), A person who loves to go out (1), Always enjoying nights out (1), Always going out in X (1), Liking going out with friends (1)

GRADUATE (3)
Mean age 63

Gender split 1 M; 2 F

A X graduate (1), A philosophy graduate (1), An economics graduate (1)

GRANDAUGHTER (14)
Mean age 20
Gender split 1 M; 13 F

A grandaughter (10), A granddaughter (4)

GRANDMOTHER (8)
Mean age 61.25
Gender split 8 F

A grandmother (5), A nana (1), Grandma (1), Grandmother (1)

GRATEFUL (2)
Mean age 38.5
Gender split 2 F

Grateful (1), Grateful to God (1)

GYM (9)
Mean age 19.78
Gender split 1 M; 8 F

A gym attender (1), A gym goer (1), A gym member (1), Member at the gym (1), A member of a gym (1), A member of the gym (2), A person who goes to the gym (1), Gym member (1)

HAIR (8)
Mean age 22.5
Gender ratio 2 M; 6 F

A brunette (1), A redhead (2), Blonde (3), Dark-haired (1), Long-haired (1)

HAPPY (43)
Mean age 26.81
Gender split 4 M; 39 F

A happy person (5), A very happy person (1), Fairly happy about my life but want to achieve more (1), Generally happy (1), Happy (25), happy about my previous professional life (1), Happy all of the time (1), Happy for things I've achieved (1), Happy go lucky (1), Happy in company of people (1), Happy most of the time (1), Happy with my family (1), Happy, confident, not shy AT ALL, even-tempered, out-going, always seeking knowledge, atheist (BHA member), am lucky with long-lasting friendships (1), Normally v happy and full of life (1), Usually happy (1)

HARD WORKING (34)
Mean age 23.56
Gender split 4 M; 30 F

A hard worker (5), A hardworking person (1), A hardworker (2), A person who studies long hours (1), A person who works hard (1), Hard working (12), Hardworking (11), Quite a hard worker (1)

HEALTH (9)
Mean age 23.44
Gender split 2 M; 7 F

Asthmatic (1), Health conscious (1), Healthy (4), On a diet (1), Reasonably unhealthy with food and exercise (1), The survivor of a bad accident (1)

HEIGHT (10)
Mean age 19.9
Gender split 3 M; 7 F

Fairly short (1), Medium height (1), Quite tall (1), Short (1), Small (3), Small in height (1), Tall (2)

HELPFUL (11)
Mean age 35.18
Gender split 2 M; 9 F

A helper (2), Good at helping people (1), Helpful (6), Willing to help (1), Willing to help anybody (1)

HISTORY (2)
Mean age 49
Gender split 2 F

A history buff (1), An interested participant in history (1)

HOME OWNER (2)
Mean age 22.5
Gender split 2 F

A home-owner (1), A house owner (1)

HONEST (12)
Mean age 35.58
Gender split 4 M; 8 F

Honest (12)

HOROSCOPE (5)
Mean age 24.4
Gender split 5 F

A cancerian (1), A gemini (1), A virgo (1), capricorn (1), taurus (1)

HOUSEMATE (2)
Mean age 19
Gender split 1 M; 1 F

A housemate (1), Living in a house of 70 people (1)

HUMOROUS (3)
Mean age 33.67
Gender split 2 M; 1 F

Humourous (2), Strangely humourous (1)

HUSBAND (10)
Mean age 56.3
Gender split 10 M

A husband (8), A husband and father (2)

IMPATIENT (3)
Mean age 20.33
Gender split 3 F

Impatient (2), Quite impatient (1)

INDEPENDENT (14)
Mean age 29.71

Gender split 2 M; 12 F

An independent person (1), Completely independent (1), Independent (9), Independent, intelligent, adaptable, love travel (1), More independent (1), Very independent (1)

INDIVIDUAL (2)
Mean age 33.5
Gender split 1 M; 1 F

An individual (1), Individual (1)

INQUISITIVE (2)
Mean age 42
Gender split 1 M; 1 F

Inquisitive (2)

INSECURE (5)
Mean age 47.4
Gender split 1 M; 4 F

Insecure (3), Socially insecure (1), Sometimes insecure (1)

INTELLIGENT (15)
Mean age 39
Gender split 4 M; 11 F

Intelligent (15)

INTERESTED (8)
Mean age 30.63
Gender split 2 M; 6 F

Intensely interested in history of, art, literature, science, the lot (1), Interested (1), Interested in people (1), Interested in psychology (2), Interested in the study of the mind (1), Interested in the world (2)

INTERESTING (3)
Mean age 18.67
Gender split 1 M; 2 F

Interesting (3)

INTERNET (2)
Mean age 21.5
Gender split 2 F

Addicted to the internet (1), One who spends a lot of time on the internet (1)

INTROVERT (11)
Mean age 44.64
Gender split 2 M; 9 F

An introvert (7), Introvert (3), Introverted (1)

IRRITABLE (3)
Mean age 36.67
Gender split 1 M; 2 F

Irritable (1), Irritated (1), Really irritable when tired (1)

KIND (12)

Mean age 25.92
Gender split 3 M; 9 F

A kind person (1), A kind, sensitive person (1), Kind (9), Kind natured (1)

LACKING COMMON SENSE (2)
Mean age 21
Gender split 2 F

Lacking in common sense (1), Rubbish at common sense (1)

LACKING CONFIDENCE (6)
Mean age 49.67
Gender split 1 M; 5 F

A person who lacks self confidence (1), Currently lacking confidence due to bullying at work (1), Lacking confidence (1), Lacking in self confidence (1), Not confident (1), Not very confident in myself (1)

LACKING KNOWLEDGE (2)
Mean age 39.5
Gender split 2 M

Not knowledgeable (1), Rubbish at general knowledge (1)

LAID BACK (10)
Mean age 22.4
Gender split 3 M; 7 F

Laid back (8), Quite laid back (1), Strangely laid back (1)

LANGUAGES (7)
Mean age 24.71
Gender split 2 M; 5 F

A chinese enthusiast (1), Able to speak Polish (1), Bad at English (1), Bilingual (1), Into sign language (1), Keen on learning languages (1), Keen on linguistic accuracy (1)

LAZY (19)
Mean age 24.16
Gender split 3 M; 16 F

A bit lazy (2), Lazy (15), Quite lazy in the mornings (1), Very lazy (1)

LEADER (2)
Mean age 21
Gender split 2 M

A leader (2)

LEARNING (7)
Mean age 34.71
Gender split 2 M; 5 F

A keen learner (1), Always learning (1), An enthusiastic learner (1), Constantly learning (1), Eager to learn (1), Keen to learn (1), Willing to learn (1)

LIFE LOVER (2)
Mean age 38.5
Gender split 1 M; 1 F

A lover of life (1), A person who enjoys life’s gifts! (1)

LIKABLE (3)
Mean age 18.67
Gender split 1 M; 2 F
Likable (2), Well-liked (1)

LISTENER (14)
Mean age 26.5
Gender split 2 M; 12 F
A good listener (11), A listener (3)

LIVELY (4)
Mean age 19.25
Gender split 4 F
Lively (3), Reasonably lively (1)

LOGICAL (2)
Mean age 41
Gender split 2 M
Logical (2)

LONELY (4)
Mean age 39
Gender split 1 M; 3 F
Lonely (4)

LONER (2)
Mean age 54
Gender split 1 M; 1 F
A bit of a loner (1), A loner (1)

LOUD (9)
Mean age 19.33
Gender split 2 M; 7 F
A loud person (2), Loud (7)

LOVE (10)
Mean age 31.9
Gender split 2 M; 8 F
A lover (1), In love (1), Loved (2), Loving (4), A lover (1), A loyal and passionate lover (1)

LOYAL (13)
Mean age 28.23
Gender split 2 M; 11 F
Loyal (12), Loyal to others (1)

LUCKY (4)
Mean age 20
Gender split 3 M; 1 F
Lucky (3), Lucky to live in X (1)

MARRIED (6)
Mean age 57.33
Gender split 2 M; 4 F
Happily married (2), Married (4)

MATHS (3)
Mean age 18
Gender split 2 M; 1 F
Good at maths (2), OK at maths (1)

MATURE (2)
Mean age 19
Gender split 1 M; 1 F
Mature (2)

ME (2)
Mean age 30
Gender split 2 M
Me (1), What I am (1)

MEMORY (2)
Mean age 39.5
Gender split 2 M
Good at remembering things (1), Someone who has a good recall of life events (1)

MESSY (7)
Mean age 19.43
Gender split 2 M; 5 F
Messy (7)

MISSING (2)
Mean age 19
Gender split 2 F
Missing a lot of people (1), Missing my dogs! (1)

MODEST (2)
Mean age 32.5
Gender split 1 M; 1 F
Modest (2)

MONEY (8)
Mean age 28.25
Gender split 3 M; 5 F
A person who spends a lot of money (1), A spender (1), Broke (1), Careful with money (1), Financially responsible (1), Not sensible with my money (1), Poor with managing money (1), Spending too much money on beer (1)

MOODY (2)
Mean age 21
Gender split 2 F
Moody (2)

MOTHER (53)
Mean age 51.79
Gender split 53 F

X, X and X's Mum (1), A good mother (1), A mother (42), A mother and grandmother (1), A mother, daughter and wife (1), Mom & granny (1), Mother (3), Mother and Grandmother (1), Mother and grandmother, and love my home as well as travelling and making - and keeping -new friends (1), Mother and Wife (1)

MOTIVATED (7)
Mean age 22.43
Gender split 2 M; 5 F

Motivated (3), Self-motivated (2), Self motivated (2)

MUSIC FAN (12)
Mean age 23
Gender split 5 M; 7 F

A big fan of music (1), A music fanatic (1), A music lover (6), A fan of music (1), Interested in music (1), Into jazz (1), Very fond of music (1)

MUSIC PLAYER (13)
Mean age 20.46
Gender split 7 M; 6 F

A drummer (1), A guitar player (1), A Guitarist (2), A keyboard player (1), A musician (5), A piano player (3)

MUSICAL (3)
Mean age 34
Gender split 1 M; 2 F

Musical (3)

NAÏVE (2)
Mean age 23
Gender split 2 F

Naïve (2)

OWN NAME (4)
Mean age 29.75
Gender split 2 M; 2 F

(Own name) (4)

NATIONALITY (17)
Mean age 33.18
Gender split 4 M; 13 F

An international hybrid (1), British (3), Canadian (1), English (2), French (1), From Latvia (1), Irish (1),Irish descent (1), Irish, with many funny and traumatic tales of the troubles (1), Part Danish (1), Part-Lithuanian (1), Phillipines (1), Polish (1), Swedish (1)

NICE (10)
Mean age 30.2
Gender split 2 M; 8 F

A nice person (2), A nice person! (1), Nice (6), Said to be too nice (1)

NIECE (5)
Mean age 20
Gender split 5 F
A niece (5)

OBSESSIVE (2)
Mean age 49.5
Gender split 2 F
An obsessive person (1), Obsessive by nature (1)

OCCUPATION (68)
Mean age 38.54
Gender split 18 M; 50 F
A bar maid (1), A bar man (1), A business owner (1), A businessman (1), A Businessman (1), A club promoter (1), A counselor (2), A DHL Driver (1), A healthcare professional (1), A lecturer (1), A legal scholar (1), A manager (1), A medievalist (1), A music teacher (1), A nurse (1), A part-time sales advisor (1), A part-time worker (1), A person who works in a bar (1), A photographer (1), A pilot (1), A professional runner (1), A psychologist (5), A psychotherapist (1), A researcher (3), A retail assistant (1), A sales assistant (1), A schoolmaster not teacher (1), shop assistant (1), A Soldier (1), A speech pathologist (1), A support worker (1), A teacher (4), A telephone interviewer (1), A tennis coach (1), A therapist (1), A university law lecturer (1), A waitress (1), A worker in a hairdressers (1), An academic (1), An accountant (1), An administrator (1), An Air Hostess (1), An educationalist (2), An employed professional (1), An employee (3), An employer (1), An entrepreneur (1), Educationist (1), Employed at school (1), Employee (1), Sandwich artist (1), Secretary to a service organization (1), Self employed (1), Self-employed (1), Working at M&S (1)

ONLY CHILD (6)
Mean age 32.83
Gender split 2 M; 4 F
A single child (1), An only child (5)

OPEN (4)
Mean age 22
Gender split 4 F
Open (1), Open once you get to know me (1), Open to challenges (1), Very open (1)

OPEN MINDED (5)
Mean age 25.8
Gender split 2 M; 3 F
Open minded (4), Very open minded (1)

OPINIONATED (2)
Mean age 35.5
Gender split 1 M; 1 F
Opinionated (2)

OPTIMISTIC (41)
Mean age 23.95
Gender split 9 M; 32 F
An optimist (5), An optimistic person (2), Optimistic (30), Optimistic most of the time (1), Optimistic sometimes (1), Optimistic, always wear a smile (1), Rather optimistic (1)

ORGANISED (11)
Mean age 24.64
Gender split 11 F

A good organiser (1), An organiser (1), Fairly organised (1), Organised (8)

OUT GOING (19)
Mean age 22.53
Gender split 5 M; 14 F

Out going (19)

OUTDOORS (9)
Mean age 46.78
Gender split 4 M; 5 F

A nature lover (1), A fan of hiking (1), A lover of the countryside (1), A lover of the outdoors (1), A lover of the outdoors (1), A nature-loving person (1), An appreciative observer of nature (1), An out of doors person (1), Happy taking long country walks (1)

OUTSPOKEN (3)
Mean age 33.67
Gender split 3 F

Outspoken (3)

PARENT (2)
Mean age 56.5
Gender split 2 M

A parent (1), Parent/spouse (1)

PARTY ANIMAL (2)
Mean age 19.5
Gender split 1 M; 1 F

A party animal (2)

PASSIONATE (5)
Mean age 34.6
Gender split 1 M; 4 F

Passionate (3), Passionate about psychology (1), Passionate about the emergence of emotional intelligence (1)

PATIENT (5)
Mean age 41.8
Gender split 2 M; 3 F

Patient (5)

PERFECTIONIST (6)
Mean age 33.17
Gender split 2 M; 4 F

A perfectionist (6)

PERFORMER (2)
Mean age 38.5

Gender split 2 F

A performer (2)

PESSIMISTIC (10)
Mean age 24.7
Gender split 2 M; 8 F

A pessimist (1), Generally pessimistic (1), Pessimistic (6), Pessimistic sometimes (1), Usually pessimistic (1)

PETS (4)
Mean age 18.75
Gender 1 M; 3 F

A dog owner (2), A pet owner (1), The owner of 2 guinea pigs (1)

PHOTOGRAPHY (2)
Mean age 55.5
Gender split 2 M

An amateur photographer (1), Photographer (1)

PLACE (15)
Mean age 25.2
Gender split 5 M; 10 F

A fan of X as a city (1), A Geordie (1), A Lancashireman, proud of it but not v. patriotic (1), A Londoner (1), A mancunian (1), A resident of X, X (1), A resident of X (1), From X (1), From X, born in X (1), From X (2), From X (1), From X (1), From the X (1), X born (1)

PLANNER (3)
Mean age 28.67
Gender split 3 F

A planner (2), Able to plan ahead (1)

POLITE (7)
Mean age 25.43
Gender split 1 M; 6 F

Polite (6), Polite to others (1)

POLITICS (11)
Mean age 52.09
Gender split 6 M; 5 F

A political activist (1), A socialist (5), A Thatcherite not a conservative (1), Interested in politics (2), Old labour, active in local politics (1), Pro-European (1)

POSITIVE (9)
Mean age 29.33
Gender split 9 F

A positive person (2), Determinedly positive (1), Positive (6)

PROBLEM SOLVER (3)
Mean age 43.67
Gender split 1 M; 2 F

A problem solver (2), Challenge solver (1)

PROFESSIONAL (2)
Mean age 43.5
Gender split 1 M; 1 F
Professional (2)

PROUD (4)
Mean age 31
Gender split 1 M; 3 F
Proud (1), Proud of my heritage (1), Proud of myself (1), Proud of my profession (flying) (1)

PUNCTUAL (5)
Mean age 26.8
Gender split 2 M; 3 F
Punctual (4), Usually on time (1)

QUIET (4)
Mean age 20.25
Gender split 2 M; 2 F
More quiet than loud (1), Quiet (3)

READER (11)
Mean age 39.09
Gender split 2 M; 9 F
A big reader, cross word doer, and therefore gifted procrastinator (1), A book-lover (1), A keen reader (1), A reader (5), A reader, grateful for my education (1), An avid reader (1), Fond of books (1)

REALISTIC (2)
Mean age 19
Gender split 2 F
Realistic (2)

REFLECTIVE (2)
Mean age 39.5
Gender split 2 M
Reflective (2)

RELAXED (5)
Mean age 19.2
Gender split 1 M; 4 F
Relaxed (5)

RELIABLE (11)
Mean age 33.45
Gender split 2 M; 9 F
Rather reliable (1), Reliable (10)

RELIGION (6)
Mean age 42
Gender split 2 M; 4 F
A relapsed catholic (1), Against religion (1), An agnostic (1), Animistic (1), Religious (2)

RESERVED (2)
Mean age 38.5
Gender split 2 F
Reserved (1), Reserved in nature (1)

RESPECT (2)
Mean age 37
Gender split 1 M; 1 F
Respected and well liked (1), Respectful (1)

RESPONSIBLE (2)
Mean age 18.5
Gender split 1 M; 1 F
Responsible (2)

RETIRED (7)
Mean age 64
Gender split 3 M; 4 F
A retired nursing professor (1), A retired senior teacher (1), A retired teacher (2), A semi-retired civil engineer (1), Retired (1), Retired teacher (1)

RISK (3)
Mean age 44.67
Gender split 2 M; 1 F
A risk taker (2), Risk averse (1)

SARCASTIC (5)
Mean age 20
Gender split 5 F
Sarcastic (5)

SCHOOL (3)
Mean age 51
Gender split 1 M; 2 F
A schoolboy (1), A schoolgirl (1), An ex-student of X (1)

SELF-CONSCIOUS (3)
Mean age 21
Gender split 1 M; 2 F
Conscious of how I appear to others (1), Self-conscious (1), Self conscious (1)

SELFISH (2)
Mean age 19.5
Gender split 2 F
Selfish (1), Sometimes selfish (1)

SENSIBLE (2)
Mean age 19
Gender split 2 F
Sensible (2)

SENSITIVE (9)
Mean age 33.33
Gender split 3 M; 6 F
A sensitive person (1), Quite sensitive (1), Sensitive (7)

SERIOUS (2)
Mean age 67.5
Gender split 1 M; 1 F
More serious in day to day matters (1), Serious minded (1)

SEXUALITY (3)
Mean age 31.67
Gender 1 M; 2 F
Heterosexual (2), Homosexual (1)

SHOPPING (11)
Mean age 19.91
Gender split 3 M; 8 F
A big shopper (1), A keen shopper (1), A lover of shopping (1), A shopper (4), Always buying trainers (1), Always window shopping (1), Good at buying presents (1), Shopping at X (1)

SHY (11)
Mean age 25.55
Gender split 2 M; 9 F
Quite shy (1), Shy (8), Shy around new people (1), Sometimes shy (1)

SINGING (8)
Mean age 30.5
Gender split 2 M; 6 F
A chorister (1), A fan of singing (1), A singer (2), A singer and performer (1), An awful singer (1), Bad at singing (1), Rubbish at singing (1)

SINGLE (7)
Mean age 40.86
Gender split 2 M; 5 F
Single (7)

SISTER (65)
Mean age 22.48
Gender split 1 M; 64 F
A sister (61), A step sister (1), A younger sister (1), Sister (1), The oldest sister (1)

SLEEP (2)
Mean age 20
Gender split 2 F
A sleeper (1), An early riser (1)

SMART (2)
Mean age 32
Gender split 1 M; 1 F
Smart (2)

SMILEY (3)
Mean age 19.33
Gender split 3 F

Smiley (3)

SOCIABLE (18)
Mean age 26.89
Gender split 6 M; 12 F

A social butterfly (1), A very social person (1), Sociable (14), Sociable yet at times need to be on my own (1), Social and enjoy going out (1)

SON (15)
Mean age 22.33
Gender split 15 M

A son (15)

SPELLING (3)
Mean age 18.33
Gender split 1 M; 2 F

Bad at spelling (3)

SPONTANEOUS (2)
Mean age 34
Gender split 1 M; 1 F

Spontaneous (2)

SPORTS FAN (13)
Mean age 30.54
Gender split 7 M; 6 F

A big football fan (1), A cricket fan (1), A cricket fanatic (1), Football fan (3), A MCFC supporter, with consequent high levels of anxiety (1), A regular attender at Everton (1), A supporter of Everton (1), A supporter of MCFC (1), An Everton fan (1), An Everton FC supporter (1), Sports-lover (1)

SPORTS PLAYER (70)
Mean age 21.81
Gender split 25 M; 45 F

A badminton player (3), A basketball player (2), A cricket player (2), A cricketer (1), A cyclist (1), A football player (1), A footballer (4), A good runner (1), A hockey player (1), A horse rider (1), A keen sportsman (1), A keen walker/exerciser (1), A korf ball player (1), A lacrosse player (2), A longboarder (1), A member of the football team (1), A netball player (7), A qualified advanced sailor (1), A rider (1), A rugby player (1), A runner (5), A sailor (1), A skateboarder (1), A skier (1), A snowboarder (2), A soccer player (1), A sportsman (1), A squash player (1), A swimmer (5), A tennis player (3), A ten-pin bowler (1) A wake boarder (1), A waterpolo player (1), An archer (1), Bad at football (1), Basketball player (1), Football player (1), Good at athletics (1), Good at Badminton (1), Into swimming (1), Sporty (3),Tennis player (1), Terrible at tennis but love it (1)

SPORTY (3)
Mean age 20.33
Gender split 2 M; 1 F

A sporty person (1), Fairly sporty (1), Natural sportsman (1)

STRESSED (4)
Mean age 22.5
Gender split 4 F
A stress head (1), Getting stressed easily (1), Stressed (1), Stressed by work (1)

STUBBORN (8)
Mean age 23.88
Gender split 3 M; 5 F
Quite stubborn (1), Stubborn (7)

STUDENT (70)
Mean age 22.54
Gender split 15 M; 55 F
A graduate student (1), A philosophy student (1), A politics student (1), A postgrad (1), A postgraduate (1), A psycholgist (student of) (1), A psychology student (14), A student (38), A student at X uni (1), A university student (1), An ex X student (1), An ex student of X College (1), An HPS-psychology student (1), An undergraduate student (1), At university (1), Doing Psychology Philosophy (1), On a psychology course (1), Studying at X university (1), Taking psychology at X (1), In first year of university (1)

STUDENT RESIDENCE (16)
Mean age 18.94
Gender split 5 M; 11 F
At (student residence name) (13), In (student residence name) (1), Living in (student residence name) (2)

STUDIOUS (3)
Mean age 23.33
Gender split 2 M; 1 F
Studious (3)

SUCCESSFUL (3)
Mean age 41
Gender split 3 M
Moderately successful (1), Successful (2)

SUPPORTIVE (2)
Mean age 35.5
Gender split 2 F
Supportive (2)

TALKATIVE (6)
Mean age 20
Gender split 6 F
Talkative (6)

TEAM PLAYER (3)
Mean age 27.67
Gender split 2 M; 1 F
A team player (3)

TELEVISION (4)
Mean age 19.25
Gender split 4 F

A telly addict (1), A tv watcher (2), A watcher of tv (1)

THINKING (6)
Mean age 29.83
Gender split 1 M; 5 F

A deep thinker (1), A thinker (2), A thinking person (1), Not great at thinking on the spot (1), Someone who thinks too much (1)

THINKS OF OTHERS (2)
Mean age 40
Gender split 2 F

A person who thinks of others before myself (1), Aware of others' feelings (1)

THOUGHTFUL (9)
Mean age 30.11
Gender split 3 M; 6 F

A thoughtful person (1), Extremely thoughtful (1), Thoughtful (7)

THRILL SEEKER (5)
Mean age 35.2
Gender split 3 M; 2 F

A thrill seeker (1), An adrenalin seeker (1), Thrill seeker (1), Thrill seeking (1), Thrill-seeker (1)

TIDY (5)
Mean age 23
Gender split 5 F

Tidy (5)

TIRED (4)
Mean age 19.75
Gender split 4 F

Easily tired (1), Tired (3)

TRADITIONAL (2)
Mean age 53
Gender split 1 M; 1 F

Moderately traditional (1), Traditionalist (1)

TRAVEL (25)
Mean age 32.04
Gender split 12 M; 13 F

A traveler (11), A traveller, tourist, long distance runner and walker (1), A traveller in mind (1), Fond of train travel (1), Happy travelling (1), Have been to Costa Rica (1), Interested in travel abroad (1), Interested in travelling (3), Into travelling (1), Planning to go somewhere different this summer (1), Traveller (1), Well travelled (1), Well-travelled (1)

TRUST (7)
Mean age 29.86
Gender split 2 M; 5 F

Trusting (1), Trustworthy (4), A trusting person (1), far too trusting of other peoples' motives, or perhaps more accurately, I am not “street-wise”, to my consistent loss (1)

TRUTHFUL (4)
Mean age 19.25
Gender split 4 F
Truthful (4)

UNDERSTANDING (6)
Mean age 27.33
Gender split 6 F
Understanding (5), Understanding to others (1)

UNIQUE (2)
Mean age 31.5
Gender split 2 F
Unique (2)

UNIVERSE (3)
Mean age 62.67
Gender split 2 M; 1 F
A physical part of the universe (1), A wonderer of the world and the universe (1), A child of the universe and god (1)

UNIVERSITY SOCIETY (5)
Mean age 19.2
Gender split 5 F
A member of Psyc Soc (2), A member of wing soc (1), A member of the skydiving society (1), Social secretary of ballet soc (1)

UPSET (2)
Mean age 24.5
Gender split 2 F
Easily upset (1), Upset about living away from home (1)

VEGETARIAN (3)
Mean age 21
Gender split 3 F
A vegetarian (3)

VOLUNTEER (4)
Mean age 25.5
Gender split 4 F
A millennium volunteer (1), A nightliner (1), A volunteer (1), A volunteer at a hospital (1)

WEIGHT (3)
Mean age 30
Gender split 1 M; 2 F
Medium weight (1), Overweight (1), Slim (1)

WIDOW (2)

Mean age 63
Gender split 2 F

A widow (2)

WIFE (25)
Mean age 50.96
Gender split 25 F

A ‘company wife’ (1), A faithful wife (1), A friend to my husband (1), A wife (14), A wife and mother (1), A wife and working mother (1), A wife, mother and grandmother (1), An exasperated wife (1), Wife (4)

WORKER (3)
Mean age 29.33
Gender split 1 M; 2 F

A worker (3)

WORRY (15)
Mean age 26.13
Gender split 1 M; 14 F

A worrier (11), Constantly worrying over nothing (1), Sometimes a worrier (1), Worried about everything (1), Worried about what others think of me (1)

WRITER (2)
Mean age 39
Gender split 1 M; 1 F

A writer (2)

UNCLASSIFIED (231)

An achiever, adaptable, an adult, affected by others' misfortunes, an air cadet, prone to putting others before myself, altruistic, ambiguous, easily amused, amusing, analytical, a bit of an anarchist, angry, sometimes easily annoyed, anonymous, not good with arguments, assertive, awestruck by natural beauty, a believer in free thinking, boring, bossy, brave, camper, career orientated, chairman of the council, looking periodically for changes, the representative for the (specific society charity event), a child, kid at heart, citizen of the world, working class, relatively clever, clumsy, said to be collected, a comedy fan, contemplative, a good conversationalist, cool, courteous, an avid crafter, critical, interested in Japanese culture, cultured, a poor decision maker, decisive, occasionally defensive, dependent, devious, a diffident person, diplomatic, dipsy, disabled, disillusioned, keen to do well in things, a doer, eccentric, a slightly educated person, educationally gifted, effective, elegant, a European, a failure, not really fascinated by things, fearful, fidgety, a first aider, not good at being formal, a freemason, full of life, fussy, wondering what the future holds, a gambler, computer geek, a geezer bird, genuine, giving, always happy to give advice if a person asks for it, glad to be alive, gluttonous, a go getter, good at acting differently around different people, good company, good to talk to, a grandson, great, greedy, a grown-up, a grumpy old woman, prone to guilt, gullible, right-handed, a hater of bureaucracy, homemaker, hopeful, horny, hot tempered, a person who attends house parties, a humanist, hungover, hungry, good at impersonating accents, indecisive, an indispensible member of staff (even though no one really is), an innovator, a man of integrity, intense, rather intent, INTJ, sometimes intolerant, intuitive, an amateur inventor/improviser, rather ironic, irresponsible, jovial, very judgmental, keen, very known for my expressions, good at attending all lectures, in the library all the time, light-hearted, literate, lovely, unable to leave my home un-make-upped, good at making stories, menopausal, subject to mood swings, sometimes nasty, a neat freak, negative, neighbours with my friend X, a nephew, nervous, aware that I have made no apparent lasting contribution to the benefit of man, not confrontational, non-judgmental, not very assertive for myself, but am for others, not

athletic, not a very hard worker, not too optimistic, not very organised, not very positive, not very proactive, not very quickly angry, not hoo u want me 2 b, a nut, part of the Officer Training Corps., an opportunistic person, often someone that people look to for answers, in my own little world, pacifist, sometimes paranoid, particular about things, a partner, pensive, a people person, perceptive, persevering, personable, persuasive, popular, practical, privileged, a procrastinator, questioning, quizzical, a Radio 4 listener, someone who likes raves, in a relationship, dreading writing another report, bad at revision, a good role model, romantic, a rule breaker, bound by my father's rules, satisfied, not able to say no easily, skeptical, often someone's secret keeper, very self-aware, self-contained, critical of myself, self-damming, self reliant, sensuous, sentimental, separated from the real world (e.g. non-student), man want sex, sexy, silly, simple in manner, slow to catch on to things, a smoker, part of the snow riders, too soft sometimes, in certain contexts, solitary, soulful, a recovering stammerer, spiritual, a steady character, straight forward, strong, one who does not suffer fools gladly, self-sufficient, super, sometimes superficial, sympathetic, someone who takes on too much, possibly the best, good at time keeping, tolerant, unanalytical, an uncle, undemonstrative, unhappy in myself, a service rep for a union, more concentrated on uni work, unjudgemental, unpredictable, unrealistic with goal setting, unrestrained, not subtle, upbeat, able to wait a rather long time for something, wanting to succeed, weird, well-presented, unable to whistle, a wimp, sometimes withdrawn, witty, work conscious, workaholic
